# Diagnosis of comorbid migraine without aura in patients with idiopathic/genetic epilepsy based on the gray zone approach to the International Classification of Headache Disorders 3 criteria

**DOI:** 10.3389/fneur.2022.1103541

**Published:** 2023-01-10

**Authors:** Arife Çimen Atalar, Aynur Özge, Bengi Gül Türk, Esme Ekizoğlu, Duygu Kurt Gök, Betül Baykan, Semih Ayta, Füsun Ferda Erdoğan, Seher Naz Yeni, Bahar Taşdelen, IDEM Study Group, Zuhal Yapıcı, Sibel K. Velioğlu

**Affiliations:** ^1^Department of Neurology, Istanbul Education and Research Hospital, University of Health Sciences, Istanbul, Türkiye; ^2^Department of Neurology, Algology and Clinical Neurophysiology, Mersin University School of Medicine, Mersin, Türkiye; ^3^Department of Neurology and Clinical Neurophysiology, Faculty of Medicine, Istanbul University-Cerrahpaşa, Istanbul, Türkiye; ^4^Department of Neurology and Clinical Neurophysiology, Istanbul Faculty of Medicine, Istanbul University, Istanbul, Türkiye; ^5^Department of Neurology and Clinical Neurophysiology, Faculty of Medicine, Erciyes University, Kayseri, Türkiye; ^6^Child Neurology Unit, Department of Pediatrics, Haseki Training and Research Hospital, University of Health Sciences, Istanbul, Türkiye; ^7^Department of Biostatistics and Medical Informatics, Mersin University School of Medicine, Mersin University, Mersin, Türkiye; ^8^Clinical Neurophysiology Unit, Department of Neurology, School of Medicine, Karadeniz Technical University, Trabzon, Türkiye

**Keywords:** migraine, idiopathic epilepsy, classification tree, headache, headache criteria, migraine without aura, ICHD-3 criteria

## Abstract

**Background:**

Migraine without aura (MwoA) is a very frequent and remarkable comorbidity in patients with idiopathic/genetic epilepsy (I/GE). Frequently in clinical practice, diagnosis of MwoA may be challenging despite the guidance of current diagnostic criteria of the International Classification of Headache Disorders 3 (ICHD-3). In this study, we aimed to disclose the diagnostic gaps in the diagnosis of comorbid MwoA, using a zone concept, in patients with I/GEs with headaches who were diagnosed by an experienced headache expert.

**Methods:**

In this multicenter study including 809 consecutive patients with a diagnosis of I/GE with or without headache, 163 patients who were diagnosed by an experienced headache expert as having a comorbid MwoA were reevaluated. Eligible patients were divided into three subgroups, namely, full diagnosis, zone I, and zone II according to their status of fulfilling the ICHD-3 criteria. A Classification and Regression Tree (CART) analysis was performed to bring out the meaningful predictors when evaluating patients with I/GEs for MwoA comorbidity, using the variables that were significant in the univariate analysis.

**Results:**

Longer headache duration (<4 h) followed by throbbing pain, higher visual analog scale (VAS) scores, increase of pain by physical activity, nausea/vomiting, and photophobia and/or phonophobia are the main distinguishing clinical characteristics of comorbid MwoA in patients with I/GE, for being classified in the full diagnosis group. Despite being not a part of the main ICHD-3 criteria, the presence of associated symptoms mainly osmophobia and also vertigo/dizziness had the distinguishing capability of being classified into zone subgroups. The most common epilepsy syndromes fulfilling full diagnosis criteria (*n* = 62) in the CART analysis were 48.39% Juvenile myoclonic epilepsy followed by 25.81% epilepsy with generalized tonic-clonic seizures alone.

**Conclusion:**

Longer headache duration, throbbing pain, increase of pain by physical activity, photophobia and/or phonophobia, presence of vertigo/dizziness, osmophobia, and higher VAS scores are the main supportive associated factors when applying the ICHD-3 criteria for the comorbid MwoA diagnosis in patients with I/GEs. Evaluating these characteristics could be helpful to close the diagnostic gaps in everyday clinical practice and fasten the diagnostic process of comorbid MwoA in patients with I/GEs.

## Introduction

Migraine is one of the most prevalent primary headache disorders in the general community and the second leading cause of disability (1) worldwide, with an estimated prevalence of 1.3 billion (2). In a nationwide community-based study from Turkey, the 1-year prevalence of migraine was reported as 16.4% (3), whereas, in another population-based study, the migraine incidence per year was found as 2.38% (4), reflecting the remarkable burden of this primary headache disorder on the otherwise healthy population.

Migraine is also frequent as a comorbidity in epilepsy, which is another paroxysmal and recurrent neurological disorder affecting many individuals globally (5). The prevalence of epilepsy is reported as 0.5–1% in the general population, whereas the incidence of migraine is reported ~2-fold in people with epilepsy (PWE), and the prevalence is estimated between 8 and 40% according to various studies with different study designs (6–10).

The clinical, pathophysiological, and symptomatic overlaps are held responsible at the forefront of the underlying mechanisms of this comorbidity since there is a hypothesis that both diseases are likely to be explained by cortical hyperexcitability. In addition, the fact that both migraine and epilepsy are strongly heritable disorders points out the importance of the genetic variants, leading to susceptibility to these disorders (6, 11–13). Also, the fact that both disorders are often responsive and can be treated with antiseizure medications (ASMs) is another supportive factor of the presence of common underlying mechanisms (14).

Despite the higher rates of coexisting migraine in PWE, it is still underdiagnosed/misdiagnosed due to several reasons such as the questioning of epilepsy and related symptoms with priority and ignoring and/or insufficient questioning of headache and associated symptoms in the routine clinical visits (6, 15, 16). Moreover, patients may ignore headaches and not mention their headache attacks to physicians/epileptologists, placing greater focus on their seizures (15).

In addition, the clinical headache symptomatology of the patients may not fulfill the established migraine diagnostic criteria, yet remain underdiagnosed, leading to ineffective, and non-standard treatments. As a consequence, the additional burden of migraine attacks to the seizures strongly and negatively affects the quality of life of these patients.

The criteria for migraine diagnosis are defined in the current International Classification of Headache Disorders 3 (ICHD-3) (17). These criteria aim to standardize the diagnosis of migraine to avoid misdiagnosis and optimize the treatment (18). However, it is remarkable that not all patients with migraine attacks can fulfill these diagnostic criteria sufficiently to be diagnosed as having “migraine.” Although the clinical symptomatology, phenotypic characteristics, genetic background, and associated symptoms point to a clinical diagnosis of migraine when evaluated concomitantly by a headache expert, these patients may not fulfill all the migraine diagnostic criteria and may not attain a “migraine” diagnosis in the routine clinical practice of a physician (19, 20). It is obvious that in everyday practice, not all patients with epilepsy with comorbid headaches can be evaluated by headache experts. It is crucial to improve the clinical decision-making process of comorbid migraine by other neurologists, practitioners, and epileptologists in the field, who deal with these patients to avoid suboptimal diagnosis in these special groups.

In this study, we attempted to classify patients with idiopathic/genetic epilepsy (I/GE) diagnosed with migraine without aura (MwoA) by a headache expert, into separate zones (namely, gray zones), according to their status of fulfilling all ICHD-3 criteria. We specifically focused on patients with I/GE because these epilepsies are acknowledged for their strong genetic background and possible shared underlying pathophysiological mechanisms (5). The data of a large population of adult and children/adolescent patients with I/GE with comorbid MwoA were evaluated and classified into three zones: “full diagnosis” (patients fulfilling all diagnostic criteria), “zone I” (patients missing only one criterion), and “zone II” (missing 2 diagnostic criteria) according to the ICHD-III classification system (19, 21).

Our primary aim was to determine the clinical supportive factors (covariates) in diagnosing MwoA in patients with I/GEs in everyday practice by using the zone concept and to highlight the role of clinical characteristics of headaches in the diagnostic process. Our secondary aim was to investigate the relationship between subtypes of I/GE syndromes with MwoA.

## Materials and methods

### Patient selection

The data of this study were derived and analyzed from the original dataset of a national, multicenter study, established between April 2019 and December 2020, by the contribution of 28 tertiary epilepsy outpatient centers from Turkey (IDEM study database).

**Figure 1:**
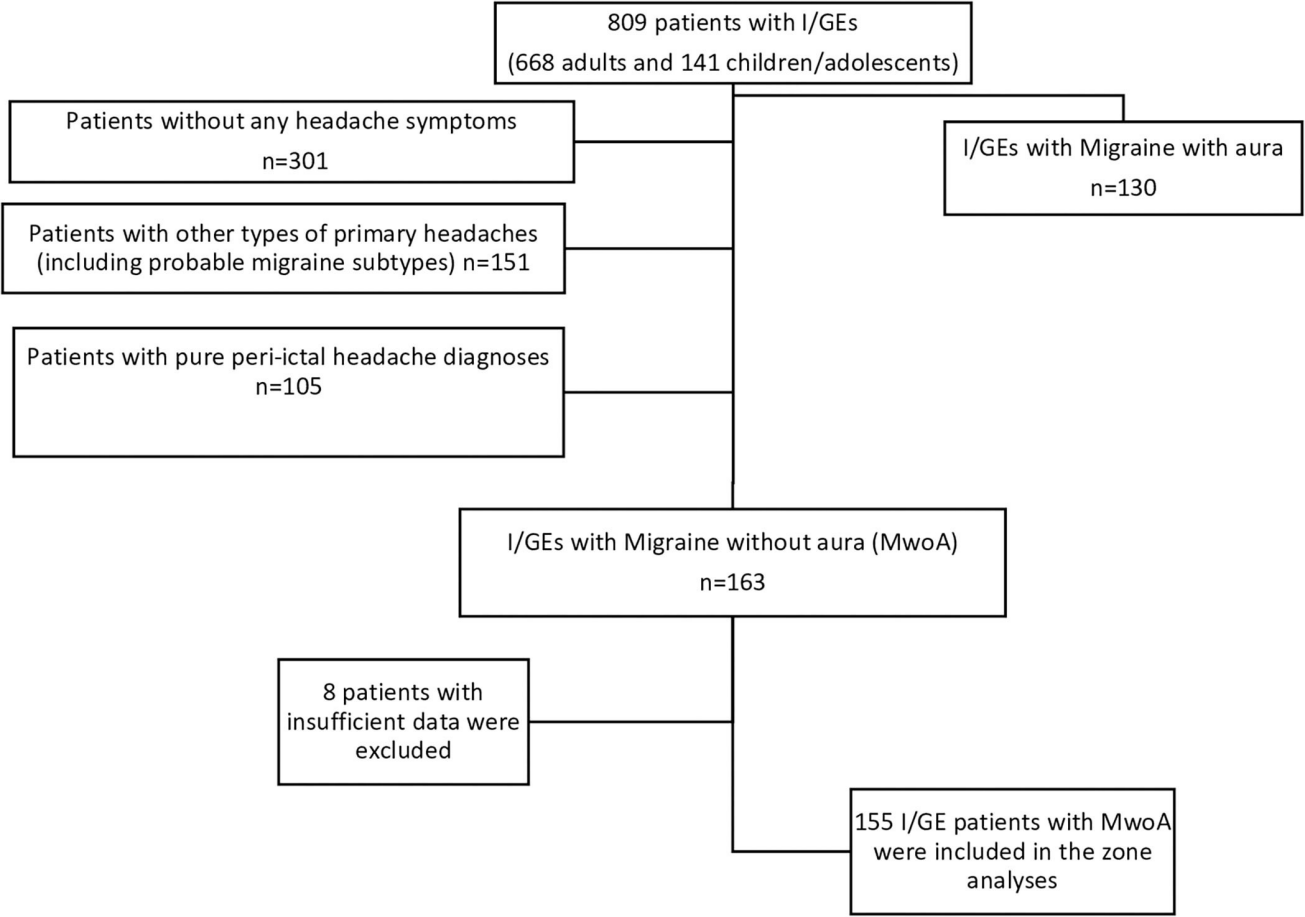
Flowchart of the study. I/GE, idiopathic/genetic epilepsy; MwA, migraine with aura; MwoA, migraine without aura; *n*, number.

From the initial dataset, which included 809 consecutive patients [668 adults (aged ≥18 years) and 141 children/adolescents (aged 6–17 years)] with a definite diagnosis of I/GEs, as defined by the International League Against Epilepsy (ILAE)-2017 criteria (22), the following patient groups were excluded:

- Patients without any comorbid headache diagnosis/headache symptoms (*n* = 301).
- Patients with other types of primary headache disorders diagnosis (e.g., tension-type headache and chronic migraine) including those with a “possible” diagnosis (*n* = 151).
- Peri-ictal headaches (headaches attributed to epileptic seizure (code 7.6; subcodes 7.6.1 and 7.6.2) (*n* = 105, one patient had both preictal type and postictal types of headaches) (refer to Figure 1 for the flowchart of the study).
- Patients with migraine with aura (MwA) were not evaluated in this study because the original study was not a prospective-diary-based study, and the interrogated details of the aura symptoms would not meet the expectations of the current study, therefore insufficient for analysis.

A total of 163 patients with an expert diagnosis of MwoA (*n* = 163) are included as seen in Figure 1.

The subgroups of idiopathic/genetic generalized epilepsies were classified as Childhood Absence Epilepsy (CAE), Juvenile Absence Epilepsy (JAE), Juvenile Myoclonic Epilepsy (JME), Epilepsy with Generalized Tonic-Clonic Seizures Alone (GTCA), and finally GGE-other (Genetic Generalized Epilepsy-other) (22, 23).

The subgroups of focal epilepsy syndromes were classified as self-limited epilepsy with centrotemporal spikes (SeLECTS) (Rolandic epilepsy, old term) and self-limited epilepsy with Autonomic Seizures (SeLEAS) (childhood epilepsy with occipital paroxysms, old term) (22, 24).

Other details of this study are reported elsewhere (21). Informed consent was obtained from all patients/legal guardians. All methods were performed in accordance with the relevant guidelines and regulations. The Ethics Committee approved the study (protocol number: 2019/32).

### Diagnostic evaluation of MwoA

The type of comorbid headache of each patient with I/GE was diagnosed and ensured by an experienced headache expert (AÖ) by reviewing the structured headache questionnaires in detail. Patients with MwoA were diagnosed by evaluating the patient's medical history, demographic and clinical characteristics, other comorbid diseases, headache symptomatology, genetic background, and headache characteristics (17), and this expert diagnosis was accepted as the “gold standard” for the diagnosis of MwoA.

Patients with MwoA were divided into three subgroups, namely, full diagnosis, zone I, and zone II, according to their status in terms of fulfilling the ICHD-3 criteria (19).

Full diagnoses implied patients with I/GE with MwoA, fulfilling the basic ICHD-3 diagnostic criteria (17), as a history of five or more headache attacks, a duration of 4–72 h (untreated or unsuccessfully treated), and showing at least two of the following characteristics: unilateral location, pulsating quality, moderate/severe pain, and aggravation by or causing avoidance of routine physical activity (e.g., walking or climbing stairs). At least one of the following symptoms should accompany during headache attacks: nausea and/or vomiting, photophobia and phonophobia, and headache not explained by any other ICHD-3 diagnosis.

The remaining patients with MwoA were classified into two zones, i.e., zone I, implying patients failing to fulfill just one of the ICHD-3 criteria, and zone II, representing patients failing to fulfill two of these basic diagnostic criteria in ICHD-3.

### Additional clinical assessments

Each patient with an expert diagnosis of MwoA was also evaluated in terms of the presence of associated clinical symptoms and properties (covariates) such as osmophobia, vertigo/dizziness, family history of headaches, family history of migraine, and autonomic features.

### Statistical analysis

Demographic and clinical variables were analyzed using the STATISTICA 13 software package by an experienced biostatistician (BT). Continuous variables are summarized as means and standard deviations and categorical variables as numbers and percentages. The diagnostic value of the clinical characteristics included among the ICHD-3-based diagnostic criteria of MwoA was evaluated in accordance with the diagnostic classification of patients as having a full diagnosis or distributed to the gray zones as I or II. Comparisons of categorical variables related to full diagnosis (providing all four diagnostic criteria), zone I (providing only three diagnostic criteria), and zone II (providing only two diagnostic criteria) were performed using the chi-square test. The mean values of body mass index (BMI) were compared using an analysis of variance (ANOVA), and the visual analog scores (VAS) of the groups were compared using the Kruskal–Wallis test. A *p* < 0.05 was considered statistically significant.

We also performed Classification and Regression Tree (CART) analysis, a non-parametric supervised learning method, to bring out the meaningful indicators when evaluating patients with I/GEs for MwoA comorbidity, using the variables (predictors) that were significant in the univariate analysis to build the CART model.

CART is used for modeling the relationships between variables for homogeneous subclasses and illustrates these relationships with a tree structure. The tree starts with the “root node.” The root nodes split to form two new nodes. Splitting continues until all “terminal nodes” are pure or contain no more than a specified minimum number of cases or objects. The purity of terminal nodes is measured using the most popular splitting measure Gini coefficient.

CART analysis is also helpful for developing guides to use in decision-making in clinical practice and elucidating the important predictors of the response without requiring assumptions between variables. Moreover, both categorical and continuous variables can be modeled together, and classification accuracy is robust for small sample sizes (25).

## Results

We evaluated 163 patients with I/GE diagnosed with an expert diagnosis of comorbid MwoA (123 female and 40 male patients; the mean age of the patients was 27.01 ± 0.83 years) by the headache expert as the gold standard, and eight of these patients were excluded due to insufficient data for statistical analysis. Finally, 155 patients (117 female and 38 male patients; the mean age of the patients was 27.12 ± 0.62 years) were classified into three groups, namely, full diagnosis and two gray zones (zones I and II). There was no statistical difference in terms of gender and age distribution between zones (*p* = 0.125 and *p* = 0.124, respectively).

The comparison of demographic and clinical properties of patients with I/GE with or without MwoA in the total study group is summarized in Supplementary Table 1.

The univariate analysis results of “full diagnosis” and zone I–II groups of patients with I/GE with MwoA are given in Table 1.

**Table 1:** The distributions of patients with idiopathic/genetic epilepsy (I/GE) with migraine without aura (MwoA) with regard to full diagnosis, zone I, and zone II.

	**MwoA**			
	**Zone II (*n* = 32)**	**Zone I (*n* = 58)**	**Full diagnosis (*n* = 65)**	***p***
A minimum of 5 headache attacks *n* (%)	16_a_ (50)	49_b_ (84.5)	65_c_ (100)	**<0.001**
Headache duration 4–72 h, *n* (%)				
<1 h	5_a_ (15.6)	10_a_ (17.2)	0_b_ (0)	
1–4 h	19_a_ (59.4)	29_a_ (50)	0_b_ (0)	**<0.001**
>4 h	5_a_ (15.6)	11_a_ (19)	30_b_ (46.2)	
>24 h	3_a_ (6.5)	8_a_ (13.8)	35_b_ (53.8)	
Unilateral location, *n* (%)	6 (19.4)	18 (31)	23 (35.4)	0.259
Throbbing pain, *n* (%)	13_a_ (40.6)	39_b_ (67.2)	53_b_ (87.5)	**<0.001**
VAS (mean ± SD)	4.969 ± 1.636^a^	6.121 ± 1.846^b^	7.123 ± 1.737^c^	**<0.001**
Increase with physical activity, *n* (%)	12_a_ (37.5)	30_a_ (51.7)	55_b_ (84.6)	**<0.001**
Nausea *n* (%)	8_a_ (25)	22_a_ (37.9)	45_b_ (69.2)	**<0.001**
Vomiting, *n* (%)	6 (18.8)	18 (31)	21 (32.3)	0.351
Photophobia, *n* (%)	19_a_ (61.3)	44_a_ (75.9)	62_b_ (95.4)	**<0.001**
Phonophobia, *n* (%)	11_a_ (34.4)	40_b_ (69)	52_b_ (80)	**<0.001**
Osmophobia, *n* (%)	6_a_ (18.8)	22_a, b_ (37.9)	29_b_ (44.6)	**0.045**
Nausea or vomiting, *n* (%)	10_a_ (31.3)	25_a_ (43.1)	47_b_ (72.3)	**<0.001**
Photophobia or phonophobia, *n* (%)	24_a_ (75)	54_b_ (93.1)	65_c_ (100)	**<0.001**
Nausea/vomiting or photophobia/phonophobia, *n* (%)	24_a_ (75)	55_b_ (94.8)	65_b_ (100)	**<0.001**
Vertigo/ dizziness, *n* (%)	5_a_ (15.6)	11_a_ (19)	30_b_ (46.2)	**0.001**
Cranial autonomic features, *n* (%)	3 (9.4)	8 (13.8)	7 (10.8)	0.791
Family history of headache, *n* (%)	20 (62.5)	32 (55.2)	38 (58.5)	0.879
Family history of migraine, *n* (%)	12 (37.5)	24 (41.4)	31 (47.7)	0.596
Comorbid systemic disease, *n* (%)	26 (81.3)	49 (84.5)	45 (69.2)	0.110
Presence of atopic dis. *n* (%)	12 (37.5)	16 (27.6)	16 (24.6)	0.411
BMI (mean ± SD)	25.418 ± 5.750	24.680 ± 4.588	23.981 ± 4.189	0.595

*p* < 0.05 is assumed statistically significant.

The identical small letters (a, b, c) in the table show the groups with no statistical difference when compared to each other.

BMI, body mass index; VAS, visual analog scale. The bold values mean “values with statistical significance”.

The univariate analysis of patients with I/GE with MwoA concerning their distribution to zones revealed that a minimum of five headache attacks, longer duration of attacks (<4 h), throbbing pain quality, higher VAS scores, increase of pain with physical activity, having nausea or vomiting, photophobia and/or phonophobia, and vertigo/dizziness was significantly higher in patients in the full diagnosis group (*p* ≤ 0.001 for each), as expected.

Interestingly, osmophobia was also statistically higher in the full diagnosis group when compared with zones I and II (*p* = 0.045). Other clinical characteristics were similar between the full diagnosis and zone groups.

**Figure 2:**
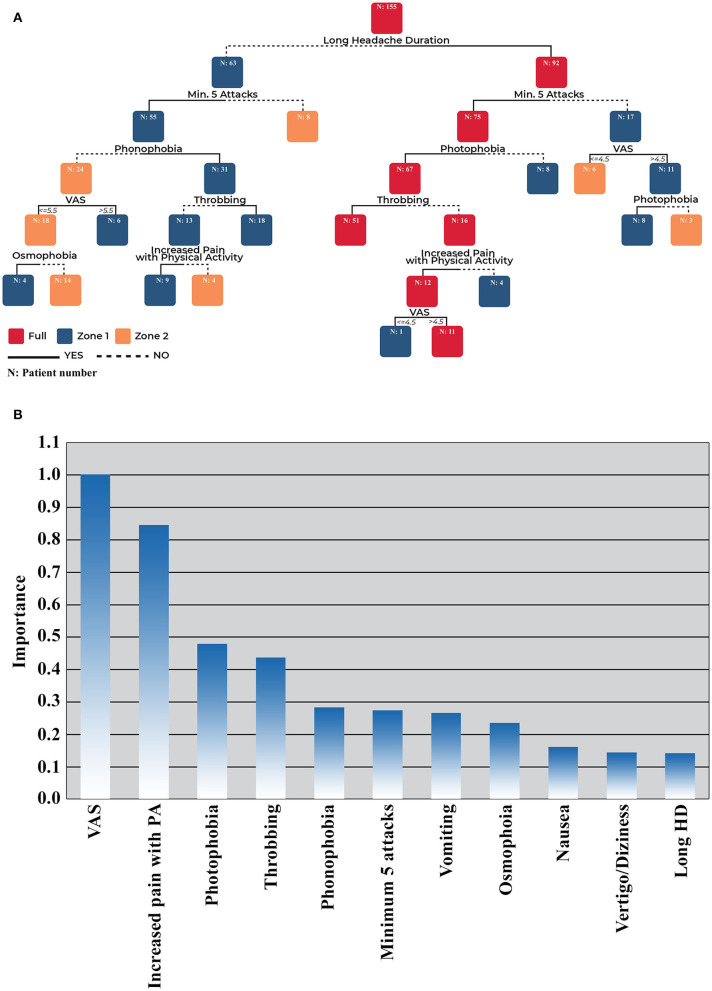
**(A)** CART analysis results of patients with I/GE with MwoA in terms of diagnostic criteria relevance*. **(B)** CART analysis relative variable importance chart. ^*^CART analysis decision tree; from top to bottom. CART, Classification and Regression Tree; HD, headache duration; I/GE, idiopathic/genetic epilepsy; Min, minimum; MwoA, migraine without aura; PA, physical activity; VAS, visual analog scale.

We performed advanced statistical analysis (CART) using the statistically significant data in univariate analysis (*p* < 0.05) for further evaluation of the predictive value of these headache criteria in terms of distributing to full migraine diagnosis, zone I or zone II subgroups (Figure 2A). A relative variable importance chart was used to determine which predictors were the most important for the branching of the tree and the development of the child nodes in the model (Figure 2B). The child nodes are sub-nodes of a node (namely, the parent node). The most important variable was VAS, and the second was increased pain with physical activity in the sub-branching of the CART tree. The model performance was good (overall accuracy = 95.12%) to classify patients into subgroups and splitting the nodes in the tree was sufficient (Gini coefficient = 0.07).

The CART analysis revealed that longer headache duration (>4 h) was the major determinant criterion in the classification of the patients into full diagnosis and zone categories. Patients with longer headaches (>4 h), a minimum of five attacks, photophobia, and throbbing headache were classified as “full.” When patients with these properties except throbbing headache were further classified, an increase of pain with physical activity and VAS scores >4.5 were also classified in the full MwoA category. Finally, 62/65 patients with MwoA in patients with I/GEs could be predicted and classified in the full diagnosis group (accuracy = 95.38%). The remaining patients who had more complicated conditions were classified into zone I and zone II subgroups.

Patients with I/GE with the following headache characteristics were likely to be predicted and categorized in zone I as having longer headache duration (>4 h), a minimum of five attacks, and photophobia but no throbbing headache and no increase in pain with physical activity. Patients without five headache attacks but with a VAS score of >4.5 were also categorized into this group. Patients with a minimum of five headache attacks, phonophobia, and throbbing headache but with a shorter headache duration (<4 h) were also grouped in zone I. If patients had these characteristics, except throbbing headache, but had an increase in pain with physical activity, they were also grouped in zone I.

Patients who had no phonophobia but had higher VAS scores (>5.5) were another group classified as zone I. Finally, patients with a minimum of five headache attacks, a shorter headache duration (<4 h), no phonophobia, and lower VAS (VAS ≤ 5.5) but with osmophobia as an associated symptom were also classified into zone I according to this analysis. CART analysis could predict 54 of the 58 patients (with an accuracy of 93.10%) as zone I.

Zone II mainly consisted of patients with shorter headache duration and infrequent headache attacks (<5 attacks). Patients with >5 headache attacks but only had phonophobia without any other headache characteristics (no throbbing headache and no increase in pain with physical activity) were also grouped as zone II. Moreover, patients with ≤ 5 headache attacks but no phonophobia, lower VAS ( ≤ 5.5), and no osmophobia were also categorized in this group. CART analysis could predict 31 of the 32 patients (accuracy = 96.88%) in the zone II group.

The epilepsy syndrome distribution of patients who fulfilled full diagnosis criteria (*n* = 62) in the CART analysis was as follows: 48.39% JME, followed by 25.81% GTCA, 16.13% JAE, 3.23% CAE, 3.23% GGE-other, and 1.61% for SeLECTS and SeLEAS. The distribution of epilepsy syndromes among all patients with MwoA and in patients classified in the full diagnosis group according to the CART analysis is given in Supplementary Figures 1A, B.

## Discussion

In this study, we highlighted the diagnostic gaps regarding comorbid MwoA diagnosis in patients with I/GEs, by using a novel “zone approach” by accepting the headache expert diagnosis as the gold standard. We found that longer headache duration (<4 h) followed by throbbing pain, higher VAS scores, increase of pain with physical activity, nausea/vomiting, and photophobia and/or phonophobia were the main distinguishing clinical characteristics of comorbid MwoA for having a full clinical diagnosis. A remarkable finding of our study was that despite not being a part of the ICHD-3 criteria, the emergence of other associated symptoms (covariates) (mainly osmophobia and also vertigo/dizziness) may support the diagnosis of comorbid MwoA in patients with I/GEs. Recognizing the comorbidity of migraine may influence the drug choice in I/GE; therefore, questioning headache existence and related symptoms in routine clinical visits could assist physicians in the correct management of these patients.

### Migraine and epilepsy continuum

Physicians may overlook headache symptoms in patients with I/GEs even though a substantial rate of migraine comorbidity (2–41.2%) has been reported in patients with epilepsy (6–10, 21, 26–29). In our multicenter study, 20.1% of all patients with I/GEs had comorbid MwoA, with the majority of these patients being diagnosed as having JME (44.1%) and GTCA (28.22%) (21).

Some possible hypotheses were suggested for this comorbidity including neuronal excitation/inhibition imbalance (in favor of cortical hyperexcitability) and cortical spreading depression (CSD) in both conditions, shared genetic susceptibility between epilepsy and migraine, neurotransmitter, and ion channel dysfunctions (14, 30, 31). All these hypotheses contribute to the concept of migraine-epilepsy as a continuum, reflecting the two different faces of shared pathophysiology (13, 14, 30, 32). The possible effects of this frequent comorbidity on the clinical presentation of epilepsy and migraine are still obscure, and studies about the outcomes are limited. We disclosed the presence of distinct headache clusters in I/GEs and the influence of this comorbidity on the clinical presentation in our previous study, and migraine-like headaches constituted one of the major headache clusters in both age groups (children/adolescents and adults) (21). Following this, we reported in this study the most important clinical factors influencing migraine diagnoses in a large group of patients with I/GEs.

### Diagnostic challenges of MwoA in a patient with I/GE

Migraine diagnosis is based on clinical aspects and is dependent on the experience of the physician dealing with the patient (15). Moreover, the physician should spare adequate time and effort to question migraine-related symptoms and associated clinical characteristics to reach an accurate diagnosis in these patients (21). The ICHD-3 criteria are the main diagnostic tool for physicians on the way to final diagnoses besides their indispensable role in the standardization and systematization of headache/migraine diagnoses among physicians (17). Despite the high reliability of fulfilling these criteria, additional information about the associated symptoms of MwoA could strengthen the diagnostic process. Therefore, especially in patients with I/GE with a MwoA comorbidity, it might be useful for the clinician in the field to question these symptoms in order to fasten the diagnostic process and increase the accuracy of headache diagnosis since these patients are usually managed by practitioners or epileptologists and not the headache experts.

Longer headache duration, one of the main characteristics of the ICHD-3 criteria (21), was also one of the main distinguishing features when classifying patients with I/GE with MwoA comorbidity into our full diagnosis or other zone categories. We observed that clinical expert diagnosis and the ICHD-3 criteria were equally compatible and valuable in diagnosing patients in the full diagnosis category. When further categorizing into zones I and II, additional clinical characteristics such as vertigo/dizziness in the univariate analysis and osmophobia and VAS values in the CART analysis were the major determinants. Accordingly, questioning these variables in clinical practice/examinations may be valuable when evaluating patients with I/GE with a comorbid headache.

Vertigo/dizziness is one of the most frequent migraine accompaniments and was reported between 30 and 54% of the patients with migraine (33–36). Although the underlying mechanisms are still not conclusively highlighted, it is speculated that vertigo and dizziness in patients with migraine may primarily be related to the functional changes in the central structures (the inner ear, brainstem, cerebellum, basal ganglia, and the hemispheres) because vestibular and trigeminal pathways overlap each other (37–39).

The trigeminal nucleus has many projections to the contralateral cortical areas (temporal, parietal, insular, and cingulate) besides its connections to the brainstem centers related to nociception such as the nucleus raphe magnus, periaqueductal gray matter, and hypothalamic areas (40–42). Moreover, there are several reciprocal connections between the vestibular system, trigeminovascular system, and nociceptive centers in the brainstem, which contribute to the modulation of the neurons in these locations (43). Another hypothesis is that certain neurotransmitters such as calcitonin-gene-related peptide (CGRP), dopamine, norepinephrine, and serotonin, which are related to migraine, might modulate the vestibular neuronal functioning (both peripheral and central) (44). Finally, genetics may play an important role in the occurrence of vestibular symptoms in patients with migraine. In many of the Genome-Wide Association Studies (GWAS), 40 loci have already been identified as associated with migraine, and it is reported that certain genes in proximity to these loci are involved in both neuronal and vascular pathways (45, 46). It is tempting to hypothesize that, in I/GEs, shared genetic factors with comorbid migraine might be effective on the emerging of vestibular symptoms. In accordance with this hypothesis, we observed higher vertigo/dizziness symptoms in patients classified in the full diagnosis group, which points to the common coexistence of this symptom in patients with I/GE with MwoA. However, we could not show vertigo/dizziness as a determinant factor in the subclassification of patients into full and gray zone categories in the advanced statistical analysis. We suggest that further studies are needed to demonstrate the significance of this symptom in diagnostic decisions of patients with MwoA in I/GE.

Osmophobia was one of the major determinants of the subclassification of patients into zones I or II in our sample. In the study by Terrin et al. (47) 45.7% of patients with MwoA attacks had associated osmophobia, and the presence of osmophobia was considered as a highly specific migrainous symptom that could be useful in differential diagnosis from tension-type headaches. In another study, 36% of patients with MwoA had accompanying osmophobia, which was reported as a valuable and remarkable symptom in the differential diagnosis (48). Similarly, in a recent study in which 444 patients with osmophobia were compared with 726 patients without osmophobia, it was reported that osmophobia was related to a longer headache duration and intensity in a large group of patients with migraine (49). Although in previous studies, osmophobia was mostly evaluated to occur together with photophobia/phonophobia as an indicator of the cortical and subcortical hyperexcitability in response to light, olfactory, and nociceptive stimuli (a generalized hypersensitivity to environmental stimuli) in migraine (48–50), we observed that the presence of osmophobia might support the clinical diagnosis of MwoA even in patients without phonophobia/photophobia. Osmophobia is accepted to be a part of the symptom spectrum related to cortical hyperexcitability in migraine, which is a possible common shared mechanism with idiopathic/genetic epilepsies. Therefore, the high frequency of this symptom in I/GEs is not surprising (51). We suggest that osmophobia could be valuable as an additional clinical criterion when evaluating patients with I/GE with headache comorbidity.

High VAS scores were another determinant factor when classifying patients into diagnostic zones in our study. In recent clinical and functional magnetic resonance imaging (f-MRI) studies, disrupted limbic system (both amygdala and hippocampus) functional connectivity to pain-related cortex regions of modulation and encoding was reported (52, 53). Resting-state functional abnormalities of the limbic system may lead to impairment of the pain process in patients with MwoA, resulting in increased pain intensity and hypersensitive response to external stimuli (52, 54). The magnitude of the neural responses in the complex network regulating pain (pain matrix) is correlated with the intensity of the perceived pain in migraine attacks (55, 56). We hypothesized that severe pain perception (higher VAS scores) might be a reflection of this impaired pain matrix and increased response to external stimuli, which could be useful in the accurate diagnosis of MwoA in clinical practice.

### Strengths and limitations

A limitation of the study may be the recall bias of the patients about the details of their migraine symptoms. However, all included patients had regular follow-ups in the contributing tertiary neurology centers, thus recall bias could be accepted as having a minimal effect on our results. Another limitation is that we could not evaluate patients with I/GE with MwA due to the missing data in the process of questioning the details of the aura criteria of the ICHD-3. In addition, in our study, disability was not taken into consideration, which is another limitation that might affect our results.

Regarding strengths, we evaluated a large number of consecutive patients with I/GEs in terms of MwoA comorbidity and gave the results of evidence-based data from this comprehensive dataset. We suggest that our results are reliable and could be helpful for clinicians and especially epileptologists when dealing with comorbid headaches in everyday clinical practice since each patient with I/GE was interviewed in detail with a standard semi-structured headache questionnaire in terms of MwoA.

## Conclusion

Migraine without aura is a frequent comorbidity in patients with I/GEs, and accurate diagnosis can sometimes be challenging given that dealing with the seizures of the patient is the priority. In routine clinical practice, screening patients with I/GE with headache in terms of longer headache duration, presence of vertigo/dizziness, photophobia/phonophobia, osmophobia, and higher VAS scores might have a supporting role in accurate MwoA diagnosis when the official criteria are not fully met. Additionally, it may speed up the diagnosis, which helps physicians in the field for targeted comorbid MwoA treatment in patients with I/GEs, reduces the burden of headaches that accompanies the burden of seizures in these patients, and improves the quality of life.

## Data availability statement

The datasets presented in this article are not readily available because of ethical and privacy restrictions. Requests to access the datasets should be directed to AA, cimenatalar@gmail.com.

## IDEM (idiopathic epilepsies and migraine) study group (affiliations at the time of the study conduct)

^a, b, c, d, e^Authors showing equal contribution are shown using an identical letter and written in alphabetical order.

Zuhal Yapıcı^1^, İpek Midi^2^, Serap Saygı^3^, Ulufer Çelebi^4^, Elif Sarıca Darol^a1^, Kadriye Ağan^a2^, Senem Ayça^5^, Sibel Gazioğlu^6^, Zeynep Vildan Okudan^b7^, Nermin Görkem Şirin^b7^, Nerses Bebek^b7^, Neşe Dericioğlu^3^, İlknur Güçlü Altun^8^, Ayşe Destina Yalçın^c9^, Reyhan Sürmeli^c9^, Oğuz Osman Erdinç^c10^, Abidin Erdal^d11^, Demet İlhan Algın^d10^, Gülnihal Kutlu^d12^, Semai Bek^d12^, Yüksel Erdal^d13^, Akçay Övünç Özön^e14^, Aylin Reyhani^e15^, Babürhan Güldiken^e16^, Barış Baklan^e17^, Bülent Oğuz Genç^e18^, Ebru Aykutlu Altindağ^e19^, Gökçen Karahan^e20^, Güray Koç^e21^, Handan Mısırlı^e22^, İbrahim Öztura^e17^, Kezban Aslan-Kara^e23^, Merve Melodi Çakar^e16^, Nur Türkmen^e18^, Onur Bulut^e17^, Ömer Karadaş^e21^, Özlem Kesim Çahin^e22^, Sevgi Ferik^e17^, Mehmet Taylan Peköz^e23^, Pınar Topaloğlu^e1^, Sibel Üstün Özek^e24^, Ülkühan Düzgün^e21^, Vildan Yayla^e25^, Yasemin Gömceli^e11^ and Zeynep Ünlüsoy Acar^e26^

^1^Department of Child Neurology, Istanbul Faculty of Medicine, Istanbul University, Istanbul, Türkiye

^2^Department of Neurology, School of Medicine, Marmara University, Istanbul, Türkiye

^3^Department of Neurology, Faculty of Medicine, Hacettepe University, Ankara, Türkiye

^4^Department of Neurology, School of Medicine, Bülent Ecevit University, Zonguldak, Türkiye

^5^Child Neurology Unit, Department of Pediatrics, Haseki Training and Research Hospital, University of Health Sciences, Istanbul, Türkiye

^6^Clinical Neurophysiology Unit, Department of Neurology, School of Medicine, Karadeniz Technical University, Trabzon, Türkiye

^7^Department of Neurology and Clinical Neurophysiology, Faculty of Medicine, Istanbul University-Cerrahpasa, Istanbul, Türkiye

^8^Department of Neurology, Dr. Lütfi Kirdar Kartal Training and Research Hospital, University of Health Sciences, Istanbul, Türkiye

^9^Department of Neurology, Umraniye Training and Research Hospital, University of Health Sciences, Istanbul, Türkiye

^10^Department of Neurology, Faculty of Medicine, Eskişehir Osmangazi University, Eskisehir, Türkiye

^11^Department of Neurology, Antalya Training and Research Hospital, Antalya, Türkiye

^12^Department of Neurology and Clinical Neurophysiology, Faculty of Medicine, Mugla Sitki Koçman University, Mugla, Türkiye

^13^Department of Neurology, Istanbul Education and Research Hospital, University of Health Sciences, Istanbul, Türkiye

^14^Department of Neurology, Yuksek Ihtisas Training and Research Hospital, Health Sciences University, Ankara, Türkiye

^15^Department of Neurology, Fatih Sultan Mehmet Education and Research Hospital, Health Sciences University, Istanbul, Türkiye

^16^Department of Neurology, School of Medicine, Trakya University, Edirne, Türkiye

^17^Department of Neurology and Clinical Neurophysiology, Faculty of Medicine, Dokuz Eylul University, Izmir, Türkiye

^18^Department of Neurology and Clinical Neurophysiology, School of Medicine, Necmettin Erbakan University, Konya, Türkiye

^19^Department of Neurology, Istanbul Florence Nightingale Hospital, Istanbul, Türkiye

^20^Department of Neurology, Bakirkoy Research and Training Hospital for Psychiatry, Neurology, and Neurosurgery, Istanbul, Türkiye

^21^Neurology Department, Gulhane School of Medicine, University of Health Sciences, Ankara, Türkiye

^22^Department of Neurology, Haydarpasa Numune Training and Research Hospital, University of Health Sciences, Istanbul, Türkiye

^23^Department of Neurology, Faculty of Medicine, Çukurova University, Adana, Türkiye

^24^Department of Neurology, Okmeydani Training and Research Hospital, University of Health Sciences, Istanbul, Türkiye

^25^Department of Neurology, Hamidiye School of Medicine, Sadi Konuk Research and Training Hospital, University of Health Sciences, Istanbul, Türkiye

^26^Department of Neurology, Haseki Training and Research Hospital, University of Health Sciences, Istanbul, Türkiye.

## Author contributions

AA: designed, analyzed, interpreted the patient data, performed data collection, and was a major contributor in writing the manuscript. AÖ: designed, analyzed, interpreted the patient data, a major contributor in writing the manuscript, and revised the first draft of the manuscript. BT and DK: data collection and interpretation of the patient data. EE and SA: designed, analyzed, and interpreted the patient data. BB and SV: designed, analyzed, and interpreted the patient data, and revised the first draft of the manuscript. FE: designed and interpreted the patient data. SY: designed and interpreted the patient data and revised the first draft of the manuscript. BT: designed and interpreted data and performed the statistical analysis. IDEM Study Group: data collection. AA and AÖ: wrote the first draft of the manuscript. All authors contributed to the study's conception and design. All authors have read and approved the final manuscript.
