# Supplementary material for: Diagnosis of comorbid migraine without aura in patients with idiopathic/genetic epilepsy based on the gray zone approach to the International Classification of Headache Disorders 3 criteria
Source: Front Neurol. 2023 Jan 10;13:1103541. doi: 10.3389/fneur.2022.1103541 (PMC9872152; doi:10.3389/fneur.2022.1103541)
Supplement: Supplementary file 1 [file Image_1.pdf]

Supplementary Figure 1. The distribution of epilepsy syndromes amongst all patients with MwoA and in patients classified in the full diagnosis group according to the CART analysis .

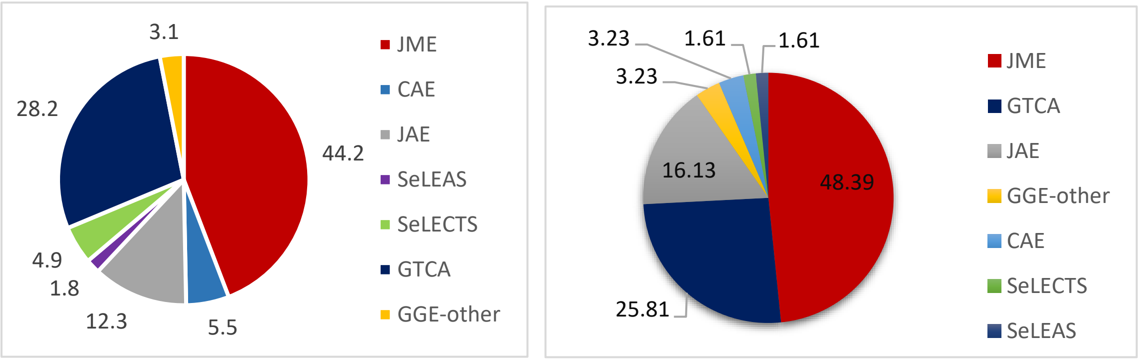

- Data expressed as percentage (%)

Abbreviations: CAE, Childhood Absence Epilepsy; GGE-other, Genetic generalized Epilepsy-other; GTCA, Epilepsy with Generalized Tonic-Clonic Seizures Alone; JAE, Juvenile Absence Epilepsy; JME, Juvenile Myoclonic Epilepsy, SeLEAS, Self- limited epilepsy with Autonomic seizures (Childhood Epilepsy with occipital paroxysms, old term); SeLECTS, Self-limited epilepsy with centro-temporal spikes (Rolandic epilepsy, old term).
