# Supplementary material for: Diagnosis of comorbid migraine without aura in patients with idiopathic/genetic epilepsy based on the gray zone approach to the International Classification of Headache Disorders 3 criteria
Source: Front Neurol. 2023 Jan 10;13:1103541. doi: 10.3389/fneur.2022.1103541 (PMC9872152; doi:10.3389/fneur.2022.1103541)
Supplement: Supplementary file 2 [file Table_1.docx]

**Supplementary Table 1.** Comparison of clinical characteristics of patient with or without MwoA

**Abbreviations:** h, hours; MwA, migraine with aura; MwoA, migraine without aura; n, number; VAS, visual analog scale; %, percentage.

|  | **MwoA** | |  |
| --- | --- | --- | --- |
|  | **(-) (n=378)** | **(+) (n=163)** | ***p*** |
| *At least 5 headache attacks* n (%) | 200 (58.3) | 130(79.8) | **<0.001** |
| *Headache duration 4-72 h,* n (%)  *<1 h*  *1-4 h*  *˃4 h*  *˃24 h* | 93(27.1)  116(33.8)  85(24.8)  49(14.3) | 19(11.7)  52(31.9)  46(28.2)  46(28.2) | **<0.001** |
| *Unilateral location*  n (%) | 93(27.0) | 47(29.0) | 0.630 |
| *Throbbing pain*  n (%) | 185(53.6) | 106(65.0) | 0.015 |
| *VAS (mean±SD)* | 5.098±2.223 | 6.166±2.016 | **<0.001** |
| *VAS ˃5*  n (%) | 200(58,3%) | 135(82,8%) | **<0.001** |
| *Increase with physical activity*  n (%) | 138(40.0) | 100(61.3) | **<0.001** |
| *Nausea* n (%) | 110(31.9) | 76(46.6) | **0.001** |
| *Vomitting*  n (%) | 80(23.2) | 46(28.2) | 0.224 |
| *Photophobia*  n (%) | 177(51.3) | 130(80.2) | **<0.001** |
| *Phonophobia*  n (%) | 177(51.3) | 109(66.9) | **0.001** |
| *Osmophobia*  n (%) | 96(27.8) | 58(35.6) | 0.078 |
| *Nausea or Vomitting*  n (%) | 129(37.4) | 84(51.5) | **0.003** |
| *Photophobia or phonophobia*  n (%) | 227(65.8) | 150(92.0) | **<0.001** |
| *Nausea / Vomitting or Photophobia / phonophobia*  n (%) | 242 (70.1) | 151(92.6) | **<0.001** |
| *Having 2 out of 4 criteria*  n (%) | 230 (61.0%) | 99(77.3%) | **<0.001** |
| *Vertigo/ Dizziness*  n (%) | 118(34.3) | 47(28.8) | 0.217 |
| *Cranial autonomic features*  n (%) | 76(22.0) | 20(12.3) | **0.007** |
| *Family history of headache*  n (%) | 164(45.7) | 95(58.3) | 0.072 |
| *Family history of migraine*  n (%) | 133(38.7) | 71(43.6) | 0.295 |
| *Comorbid systemic disease*  n (%) | 269(78.0) | 126(77.3) | 0.865 |
| *Presence of atopia*  n (%) | 93(27.0) | 45(27.6) | 0.878 |
